# Au-Coated ZnO Surface-Enhanced Raman Scattering (SERS) Substrates: Synthesis, Characterization, and Applications in Exosome Detection

**DOI:** 10.3390/chemosensors11110554

**Published:** 2023-11-05

**Authors:** Samuel Adesoye, Saqer Al Abdullah, Anjali Kumari, Gayani Pathiraja, Kyle Nowlin, Kristen Dellinger

**Affiliations:** 1 Department of Nanoengineering, Joint School of Nanoscience and Nanoengineering, North Carolina A&T State University, 2907 E Gate City Blvd, Greensboro, NC 27401, USA;; 2 Department of Nanoscience, Joint School of Nanoscience and Nanoengineering, University of North Carolina at Greensboro, 2907 E Gate City Blvd, Greensboro, NC 27401, USA;

**Keywords:** Raman spectroscopy, biosensing, exosomes, hybrid nanoparticles, zinc oxide

## Abstract

Developing a biomolecular detection method that minimizes photodamage while preserving an environment suitable for biological constituents to maintain their physiological state is expected to drive new diagnostic and mechanistic breakthroughs. In addition, ultra-sensitive diagnostic platforms are needed for rapid and point-of-care technologies for various diseases. Considering this, surface-enhanced Raman scattering (SERS) is proposed as a non-destructive and sensitive approach to address the limitations of fluorescence, electrochemical, and other optical detection techniques. However, to advance the applications of SERS, novel approaches that can enhance the signal of substrate materials are needed to improve reproducibility and costs associated with manufacture and scale-up. Due to their physical properties and synthesis, semiconductor-based nanostructures have gained increasing recognition as SERS substrates; however, low signal enhancements have offset their widespread adoption. To address this limitation and assess the potential for use in biological applications, zinc oxide (ZnO) was coated with different concentrations (0.01–0.1 M) of gold (Au) precursor. When crystal violet (CV) was used as a model target with the synthesized substrates, the highest enhancement was obtained with ZnO coated with 0.05 M Au precursor. This substrate was subsequently applied to differentiate exosomes derived from three cell types to provide insight into their molecular diversity. We anticipate this work will serve as a platform for colloidal hybrid SERS substrates in future bio-sensing applications.

## 1. Introduction

Accurate detection of biological constituents and molecules is essential to understanding molecular changes in pathophysiological conditions, which can serve as an important target for therapeutic interventions and biomarker discovery. Exosomes, which are a subclass of extracellular vesicles, are now recognized as novel mediators for cell-to-cell communication and repositories for biomarkers in various diseases, including cancer [1,2], Alzheimer’s disease [3–5], and cardiovascular disease [6,7]. To detect exosomes for diagnostic applications, surface-enhanced Raman scattering (SERS)-based methods have several unique capabilities, such as fingerprint-like identification, non-destructive analysis, easy sample preparation, low sample interference with water, high sensitivity, and multiplexing capabilities [8]. However, SERS substrates, which provide a surface to enhance the signal of localized molecules, are continually being developed to improve reliability and reproducibility and to make SERS a breakthrough technology in the biomedical space. For instance, semiconductors, which benefit from the chemical enhancement mechanism and possess unique physical properties, are continually being improved to mitigate their shortcoming of low enhancement efficiency [9]. Substitutional doping [3] and coating with noble metals, e.g., Au [4], have been explored to improve the Raman enhancement of these semiconductor materials. While substitutional doping could introduce defects for effective charge transfer, coating with noble metals provides an electromagnetic enhancement from plasmon excitation in the metal nanostructures. A strong electromagnetic enhancement is observed from noble metals since their localized surface plasmon resonance (LSPR) bands are in the visible spectral region [10], allowing for strong light absorption. For semiconductors, the LSPR peak of their valence band (VB) is centered in the UV region due to high electron density, while the LSPR peak of the conduction band is at the infrared spectra region due to low electron density [10,11]. Therefore, since LSPR dominates electromagnetic effects under visible light, plasmons in semiconductors rarely contribute to electromagnetic enhancement effects [11]. However, irregularly coating semiconductors with noble metals could harness the dual effects of electromagnetic enhancement from the plasmonic metal and chemical enhancement from the semiconductor [12–14]. When the plasmonic metal is introduced into a semiconductor, the LSPR peak is tuned to the near-infrared spectra and visible region, and hotspots (gaps between the metallic nanoparticles) are also created [11,15]. These hotspots can create a local electric field when irradiated with light to provide a larger enhancement effect [11]. In addition, this iteration provides sizeable surface areas for plasmonic metals to be attached, increasing the possibility of forming hotspots as more molecules or target analytes can interact with the substrate surface [16].

Exosomes are phospholipid bilayer-enclosed, nanosized extracellular vesicles secreted by nearly all cells in the body [2,8]. Exosomes can be isolated from body fluids, such as urine and blood, providing accessible sources for isolation and downstream detection [17]. Theoretically, exosomes from different origins have specific functional biomolecules, i.e., specific genotypes and molecular phenotypes, which can be reflected in their SERS spectra [2]. Also, the chemical composition of exosomes may depend on the physiological state of the cells they originate from and could provide a signature of specific diseases, positioning them as useful candidates in determining healthy or diseased states [17]. SERS has been used in both direct and indirect techniques for biosensing. The direct method is regarded as “label-free”, while the indirect method utilizes a Raman reporter molecule, often linked with a targeting molecule (e.g., antibody or aptamer) to target an analyte [9]. Previous studies have used the indirect SERS sensing technique to detect exosomes [8,18]; however, clinical translation is limited due to the complexity of this method. Also, direct SERS sensing has been explored for the rapid detection of exosomes derived from breast cancer [2], liver cancer [19], and lung cancer [20] cells. However, due to the ultralow concentration of several biologic samples, including exosomes, there is a need to continually develop the substrate materials to achieve low detection limits and effectively distinguish exosomes derived from different cellular origins.

In this work, zinc oxide (ZnO) was coated with different concentrations of Au precursor (HAuCl_4_) using a wet chemistry method to yield hybrid substrates applicable in SERS biosensing. This substrate was subsequently applied to differentiate exosomes isolated from neuroblastoma cells (N2a), macrophages (RAW 264.7), and breast cancer cells (MCF-7), to provide insight into molecular variability of exosomes from different cells. In this study, we sought to demonstrate the ability of hybrid SERS substrates to unravel the diversity of extracellular vesicles and provide a foundational platform for future diagnostic applications.

## 2. Materials and Methods

### 2.1. Chemicals and Reagents

Zinc acetate dihydrate was purchased from Sigma-Aldrich (St. Louis, MO, USA); HAuCl_4_ was purchased from Fisher Scientific (Muskegon, MI, USA); sodium hydroxide was purchased from Sigma-Aldrich; crystal violet (CV) was purchased from Thermo Fisher Scientific (Fair Lawn, NJ, USA). N2a, RAW 264.7; and MCF-7 cells, Dulbecco’s Modified Eagle’s Medium (DMEM), penicillin/streptomycin, and fetal bovine serum (FBS) were purchased from ATCC (Manassas, VA, USA).

### 2.2. Preparation of Au-Coated ZnO Nanoparticles

ZnO nanoparticles were synthesized using a co-precipitation method described in our previous work [21]. In brief, an equal volume of 0.1 M ZnAce and 0.2 M NaOH was added into a 100 mL round bottom flask. The mixture was magnetically stirred at 750 rpm and heated to 60 °C for 2 h. The product was extracted into a falcon tube, centrifuged at 10 Krpm for 5 min, washed with water four times, and dried in an oven for 6 h at 80 °C. To synthesize Au-coated ZnO, 100 mg of ZnO was dispersed in 10 mL water via sonification for 30 min. Next, 10 mL of HAuCl_4_ was added to the dispersed ZnO solution, stirring continuously for about 2 h at 750 rpm. The precipitate was washed with water to remove excess HAuCl_4_ from the ZnO and re-dispersed in 20 mL DI water. Finally, 0.2 mL of 1 M hydrazine hydrate was added to the solution while continually stirred [22]. Different concentrations of HAuCl_4_ (0.01, 0.05, and 0.1 M) were used in the synthesis to obtain ZnO-Au1, ZnO-Au2, and ZnO-Au3 substrates, respectively.

### 2.3. Hybrid SERS Substrate Characterization

To characterize these substrates, the morphology and size distribution of the nanoparticles were obtained using a Zeiss Auriga field-emission scanning electron microscope (FE-SEM) equipped with an energy dispersive X-ray (EDX) spectrometer and transmission electron microscopy (TEM), while ESCALAB^™^ X-ray photoelectron spectroscopy (XPS) was used to determine the elemental composition and the binding energy of the materials. Optical properties were obtained with an Agilent 3000i UV-Vis spectrophotometer, while a confocal Raman microscope (Horiba, Piscataway, NJ, USA) and i-Raman Prime portable Raman spectrometer (B&W Tek, Newark, DE, USA) equipped with a 785 nm laser source were used to acquire the Raman signal.

### 2.4. Cell Culture

Mouse neuroblastoma (N2a), breast cancer cells (MCF-7), and mouse macrophage cell line (RAW 264.7) were cultured in complete Dulbecco’s modified Eagle’s medium (DMEM) supplemented with 1% penicillin/streptomycin and 10% fetal bovine serum. The cells were incubated at 37 °C in a humidified 5% CO_2_ incubator.

### 2.5. Exosome Isolation and Characterization

The cells were exposed to a media change without FBS after reaching 80% confluency. According to the manufacturer’s protocol, exosomes were isolated from the collected culture media of N2a, MCF-7, and RAW 264.7 using a Total Exosome Isolation Kit^®^ (Thermo Fisher). In brief, the cultured media was centrifuged at 2000× *g* for 30 min to remove cells and debris. The resulting supernatant was transferred to a new tube and mixed with the kit in a 2:1 ratio, followed by centrifugation at 10,000× *g* for 60 min at 4 °C. The obtained pellet was resuspended in 50 μL of phosphate-buffered saline (PBS) and stored at −80 °C for downstream analysis and SERS testing. Exosome size and concentration were determined using a NanoSight LM10 (Malvern Panalytical, Westborough, MA, USA). The samples were diluted about 12 times and then injected into the sample chamber. Exosome size and distribution were analyzed using NTA v3.2 analytical software.

### 2.6. SERS Detection

The SERS signal of 10^−6^ M concentration of CV was acquired with the various substrates, while the SERS signal of CV concentrations ranging from 10^−2^ to 10^−8^ M was acquired with the optimal substrate. The i-Raman Prime portable Raman spectrometer is equipped with an accessory to acquire the Raman signal of liquid samples in a compatible vial. Thus, the SERS signals of the CV solutions were acquired directly with the colloidal substrate. An equal 150 μg/mL substrate volume and CV were dispersed and sonicated before acquiring the SERS signal. The acquisition time was 1000 ms at 100% laser power and an accumulation number of 3. For the exosomes, the ZnO-Au2 substrate was deposited on glass, and about 50 μg/mL exosomes were cast on the substrate and dried before acquiring the SERS signal with a confocal XplorRA Raman microscope (HORIBA Scientific, Piscataway, NJ, USA).

## 3. Results and Discussion

### 3.1. Synthesis and Characterization of Au-Coated ZnO

**Figure 1.**
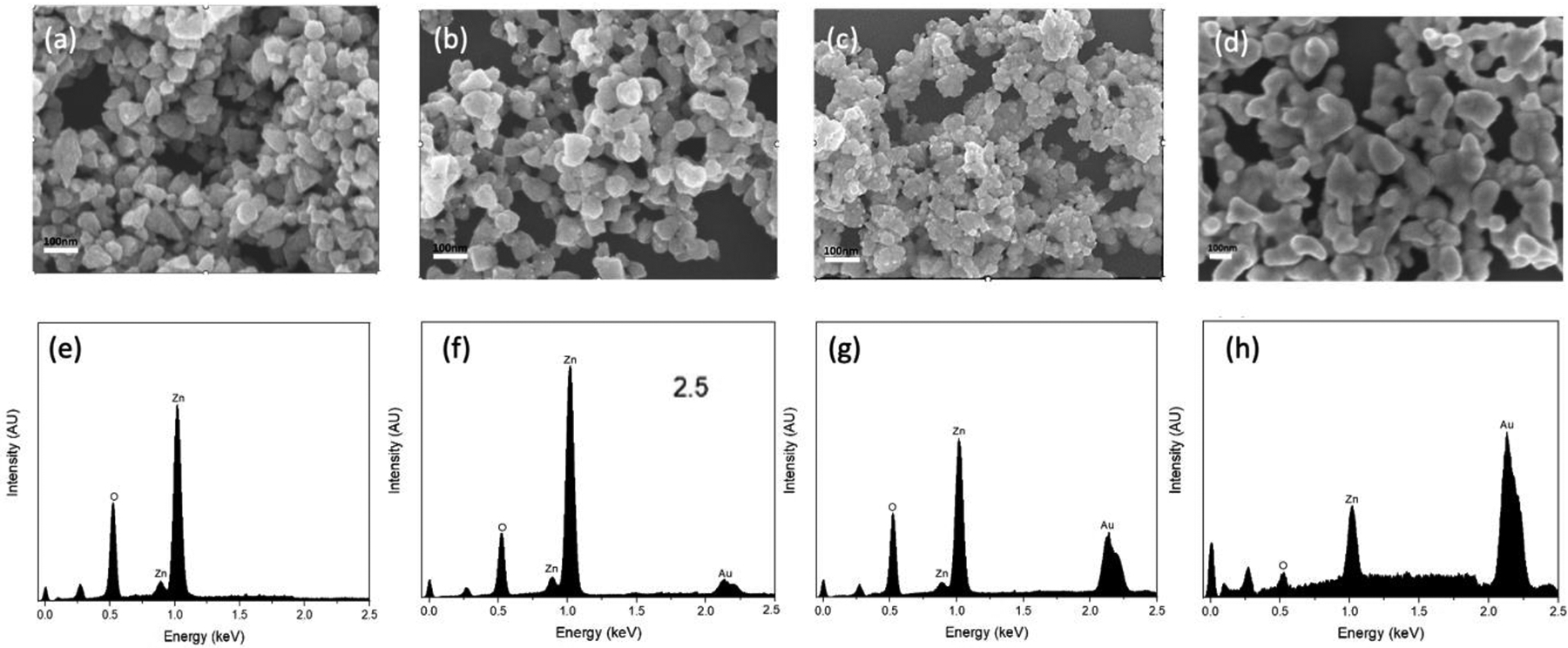
SEM images of (**a**) pure ZnO, (**b**) ZnO-Au1, (**c**) ZnO-Au2, (**d**) ZnO-Au3. EDX survey spectra of (**e**) pure ZnO, (**f**) ZnO-Au1, (**g**) ZnO-Au2, (**h**) ZnO-Au3.

**Figure 2.**
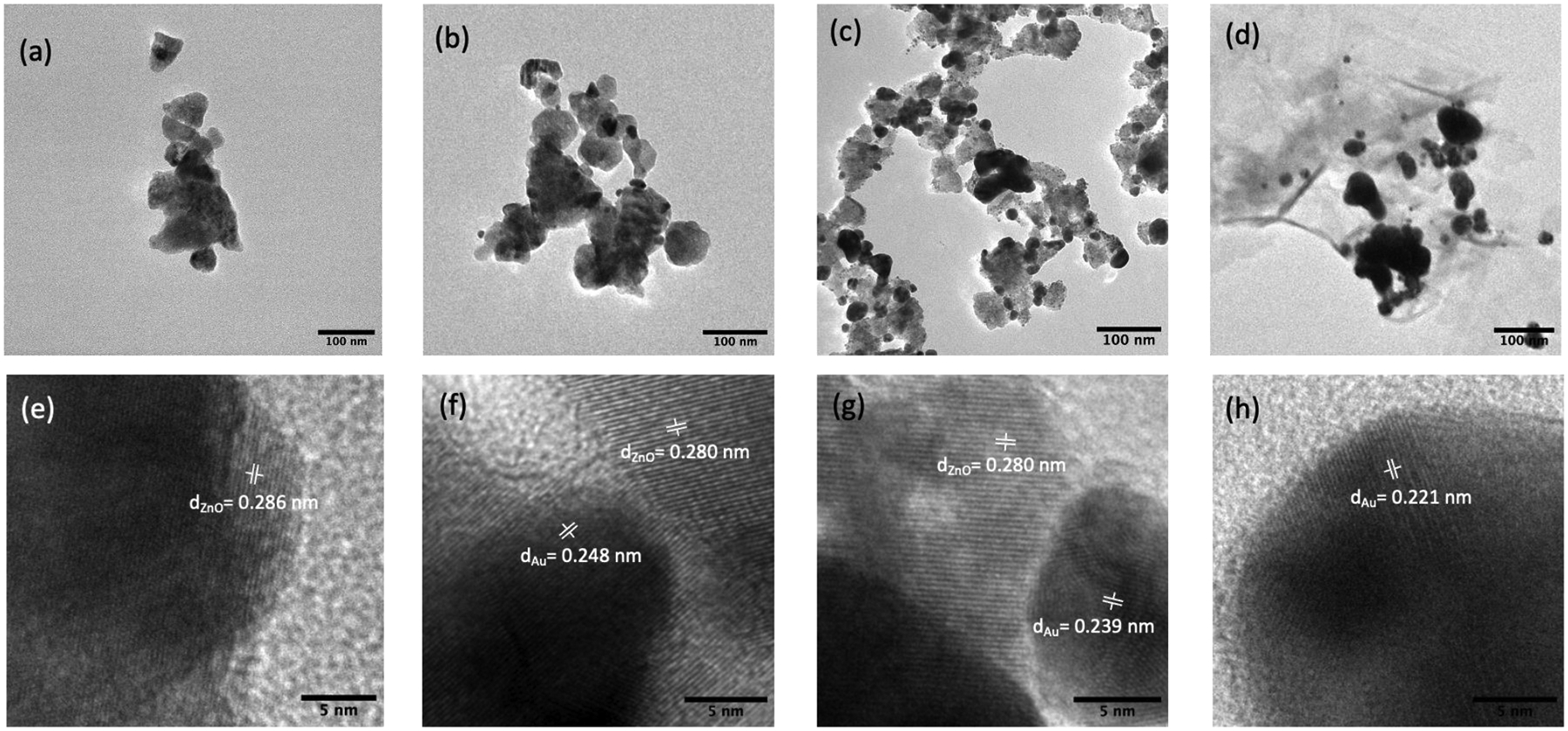
TEM images (**a**) pure ZnO, (**b**) ZnO-Au1, (**c**) ZnO-Au2, (**d**) ZnO-Au3. HR-TEM image with respective lattice spacings of (**e**) pure ZnO, (**f**) ZnO-Au1, (**g**) ZnO-Au2, (**h**) ZnO-Au3.

The morphology of the synthesized substrates and Au distribution over ZnO were investigated using FE-SEM and TEM. As shown in Figure 1a, the pure ZnO substrates had an average size of ca. 70 nm and a smoother surface compared to ZnO-Au substrates (see Figure 1b–d). Images of the ZnO-Au substrates showed scattered spherical Au particles distributed on the surface. Also, as the concentration of Au precursor increased, more Au particles were deposited on the ZnO surface. The increase in the deposition of Au particles was also confirmed with EDX analysis Figure 1e–h. These substrates were further analyzed using TEM, as shown in Figure 2a–h. For the ZnO-Au2 substrate depicted in Figure 2c, Au nanoparticles are indicated by darker spots, and the ZnO support is shown in a less intense color. In addition, there was a uniform dispersion of smaller Au nanoparticles over the entire ZnO surface, with sizes of Au nanoparticles ranging between 20 and 30 nm and a spacing of approximately 5 nm between adjacent Au nanoparticles. Fringes are also visible throughout the crystal plane, with the observed lattice fringes of width 0.24 nm and 0.28 nm d-spacing, which can be associated with (111) crystal plane of Au [22–24] and (100) crystal plane of ZnO [25], respectively. Figure 2b shows that less Au was deposited on the ZnO, thus forming a larger nanogap. On the other hand, Figure 2d showed the disappearance of nanogaps, which could be due to the high concentration of Au precursors. Noble metals ranging between a few tens of nanometers have been shown to amplify the local electromagnetic field, thereby enhancing the SERS signal [23,26]. Additionally, a local electric field in the confined junctions, often referred to as “hotspots”, could be huge when the gap between the Au nanoparticles is less than 10 nm due to strong plasma resonance when irradiated by the laser [23,27], which may explain the superior SERS performance of ZnO-Au2 compared to other substrates as detailed in the next section.

**Figure 3.**
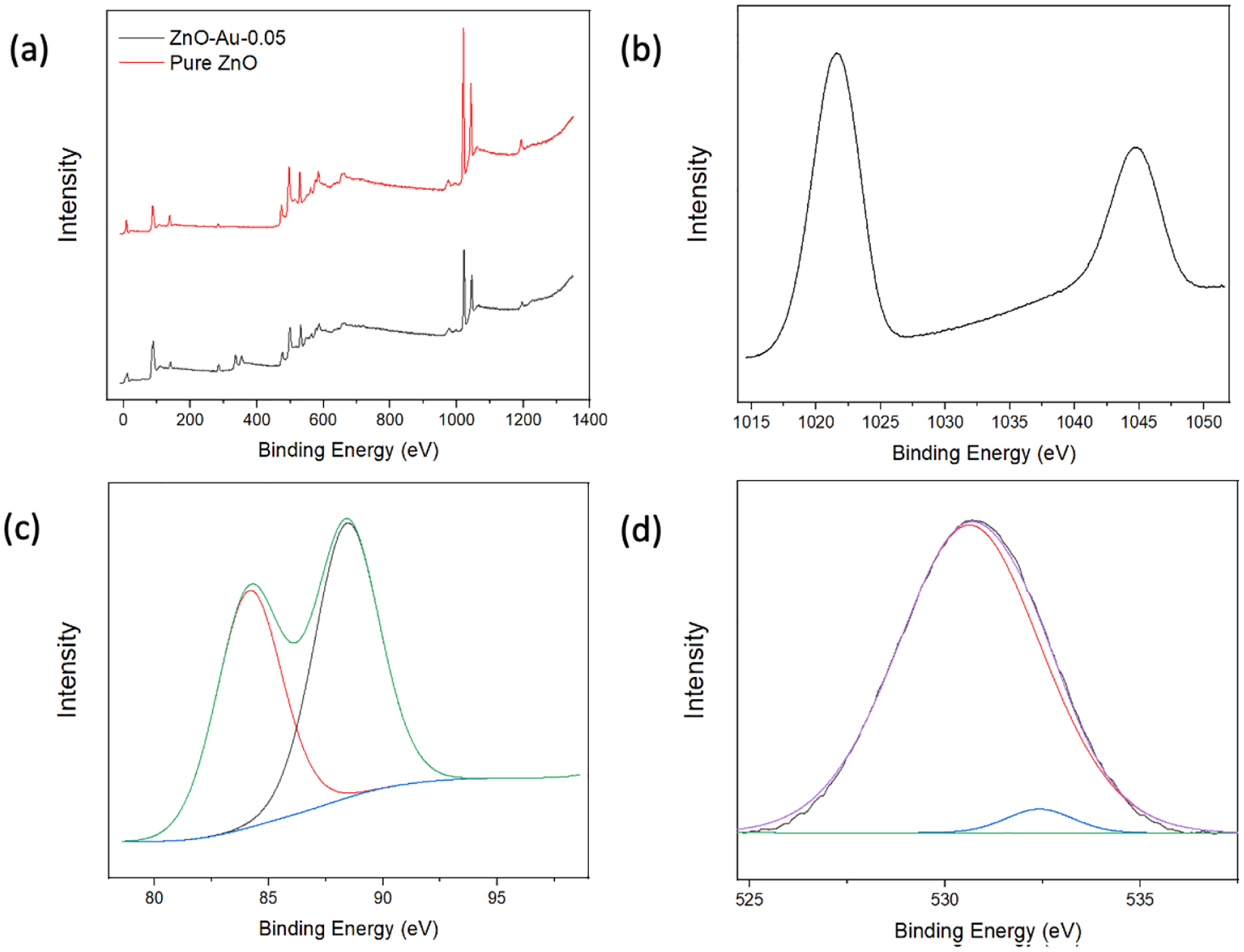
(**a**) XPS full scan of pure ZnO and ZnO-Au2. (**b**) High-resolution Zn scan of ZnO-Au2. (**c**) High-resolution Au Scan of ZnO-Au2. (**d**) High-resolution O scan of ZnO-Au2.

XPS analysis was employed to characterize the elemental composition and study the chemical states of the elements present in the substrates. As shown in Figure 3a, the XPS scans indicate the presence of Zn and O in all samples and Au in the coated samples. Figure 3b shows a high-resolution Zn2p scan with peaks at 1021.6 eV and 1044.7 eV, which can be attributed to the binding energy lines of Zn2p^3/2^ and Zn2p^1/2^, and a 23.1 eV separation indicating the Zn atom is in the +2-oxidation state. The high-resolution XPS spectra of Au show peaks at 84.2 eV and 88.4 eV corresponding to Au4F_7/2_ and Au4F_5/2_, respectively [23,24,28]. The high-resolution spectrum of O can be split into two Gaussian peaks, as shown in Figure 3d; the peak centered around 530.62 eV corresponds to O^−2^ on the wurtzite structure of Zn^2+^, while the peak centered around 532.42 eV can be attributed to oxygen vacancies [21]. Overall, the XPS analysis indicated that Au was associated with ZnO, which could thereby provide the electromagnetic contribution towards the enhancement of Raman signal in subsequent applications.

**Figure 4.**
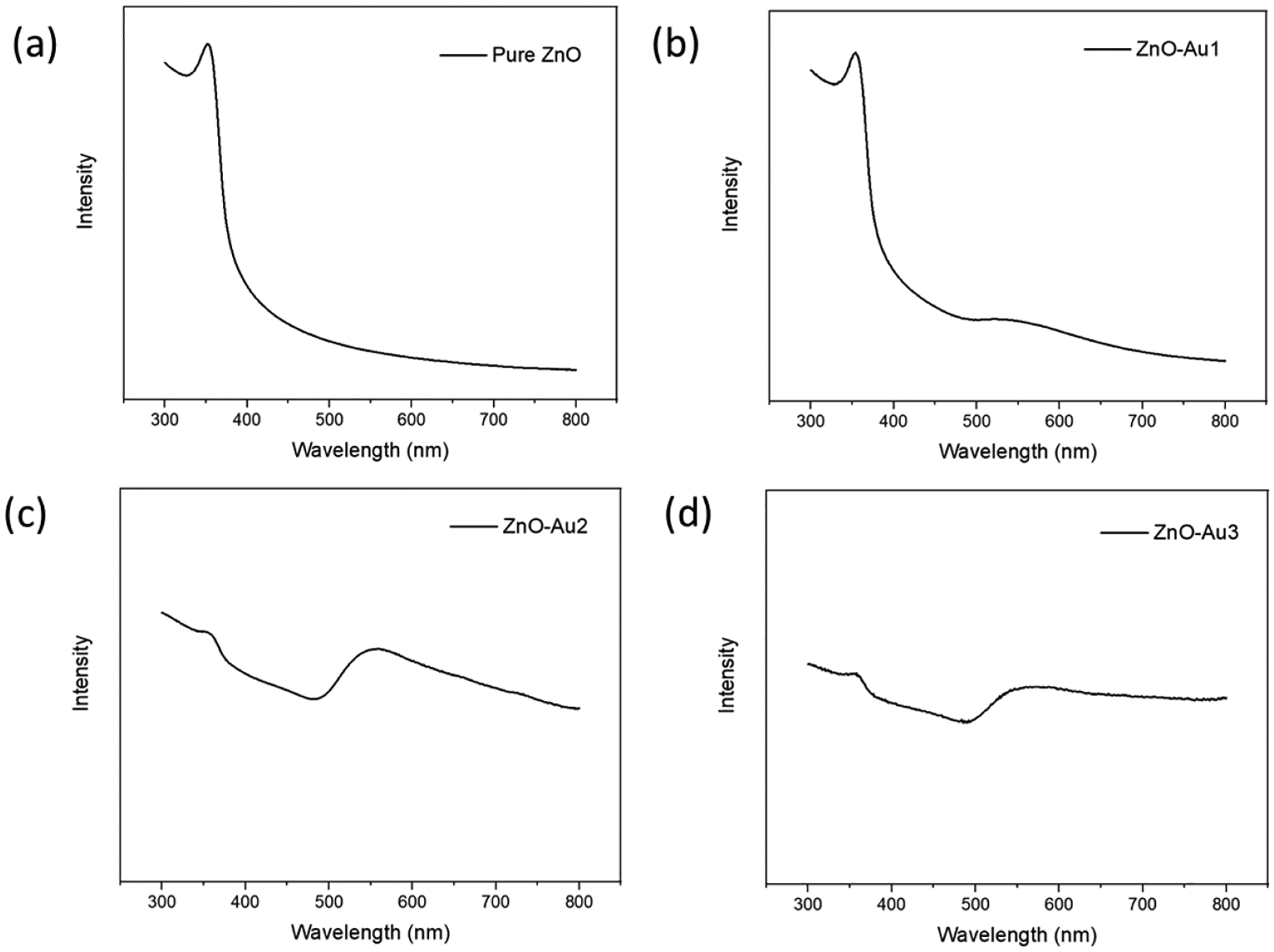
UV-VIS spectra of (**a**) pure ZnO, (**b**) ZnO-Au1, (**c**) ZnO-Au2, (**d**) ZnO-Au3.

The synthesized substrates’ UV–visible absorption spectra were obtained to determine their optical properties. As shown in Figure 4, all spectra showed an absorption band at 358 nm, corresponding to ZnO [21]. For the ZnO-Au samples, a broad band was observed at 560 nm, corresponding to Au nanoparticles and thereby indicating that the ZnO-Au substrates are active in both the ultraviolet and visible light region [22]. In addition, the ZnO-Au substrates synthesized with a higher concentration of the Au precursor, i.e., ZnO-Au2 and ZnO-Au3, showed a low absorbance intensity of the ZnO peak, which could have resulted from an increased amount of Au deposited on ZnO.

### 3.2. SERS Performance

**Figure 5.**
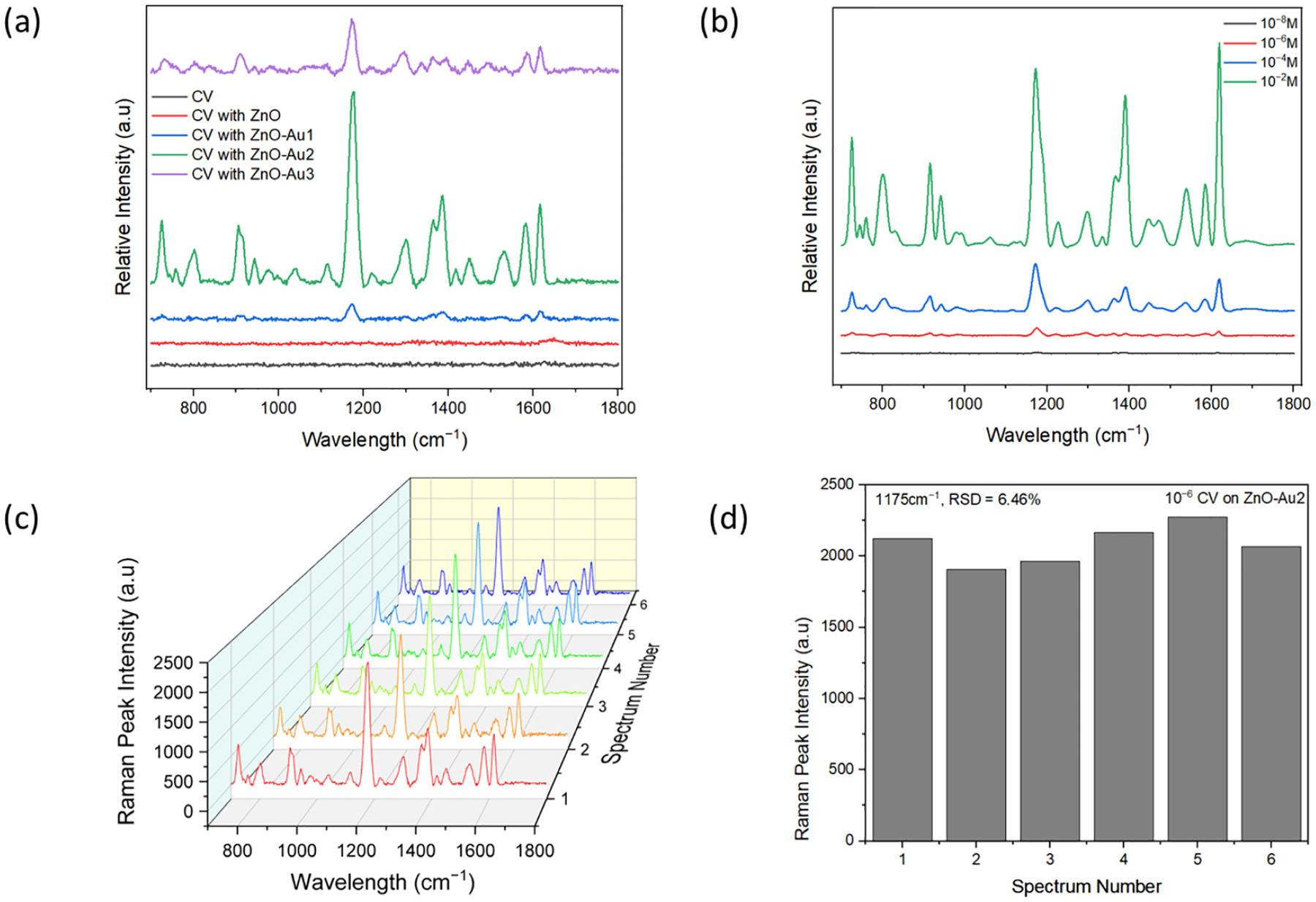
(**a**) SERS spectra of 10^−6^ M crystal violet (CV) on pure ZnO and ZnO-Au substrates. (**b**) SERS spectra of different concentrations of CV on the optimal substrate (ZnO-Au2). (**c**) SERS spectra of six random measurement of 10^−6^ M CV on optimal substrate. (**d**) SERS peak intensity at wavelength 1175 cm^−1^ of the six random measurements of 10^−6^ M CV on optimal substrate.

To demonstrate the SERS performance of the ZnO-Au substrates synthesized using different concentrations of Au precursor, 10^−6^ M CV was used as a probe molecule. Notable peaks observed were 801, 916, 941, 1175, 1365, 1539, 1584, and 1619 cm^−1^, corresponding out of plane C–H bending, C–C stretch (in-ring), C–C stretch (in-ring), in-plane C–H bending, C_center_–C stretch, C_ring_–N stretch, C–C stretch (in-ring), and C–C stretch (in-ring), respectively [29–31]. As shown in Figure 5a, with increasing concentrations of Au precursor, the SERS intensity of CV increased, then subsequently decreased. The lower signal intensity observed with a low concentration of Au precursor could have resulted from fewer Au nanoparticles deposited on the ZnO (see Figure 2b), which could have led to the formation of larger nano-gaps. On the other hand, the reduced signal intensity observed with a high concentration of Au precursor could have resulted from the agglomeration of Au nanoparticles, leading to the disappearance of nano-gaps (see Figure 2d). However, with an appropriate concentration of Au precursor, there was a uniform deposition of Au nanoparticles, potentially forming dense “hotspots” and resulting in a higher signal enhancement. Dense nanogaps were also confirmed with TEM images (Figure 2b). When different concentrations of CV were tested with the optimal substrate (ZnO-Au2), it was observed that the signal intensity increased with increasing concentrations of CV, and a 10^−8^ M limit of detection of CV was achieved. The reproducibility of the SERS signal was tested by acquiring six random spectra of 10^−6^ M of CV with the optimal substrate. As shown in Figure 5c, the SERS spectra showed a high degree of similarity. For the 1175 cm^−1^ SERS peak, the relative standard deviation (RSD) of the peak intensity was evaluated to be 6.46% (see Figure 5d), indicating the uniformity of the substrate and reproducibility of the SERS signal.

Using the peak at 1175 cm^−1^, the average enhancement factor (AEF) was calculated from the equation:

(1) $$\text{AEF}=\frac{I_{\text{SERS}}}{I_{\text{Raman}}}\times \frac{C_{\text{Raman}}}{C_{\text{SERS}}}$$

where *I*_*SERS*_ and *I*_*Raman*_ are intensities of the peak acquired with substrate and without the substrate, respectively, while *C*_*SERS*_ and *C*_*Raman*_ are the lowest concentrations of CV that yielded an observable peak for SERS and Raman detection [32–35]. The values obtained for *I*_*SERS*_ and *I*_*Raman*_ are 3023 and 1037.67, while the values of *C*_*SERS*_ and *C*_*Raman*_ are 10^−8^ M and 10^−4^ M, respectively. From these values, the calculated AEF is 2.9 × 10^4^. The obtained AEF is higher than values obtained with previous methods employed to coat Au on ZnO [36,37].

It is well established in the literature by both experimental and theoretical studies that SERS enhancement results from both electromagnetic and chemical effect [10,15,28,35,38–41]. Electromagnetic enhancements are attributed to plasmon excitation in metal nanoparticles functioning as a substrate, while chemical enhancements are more broadly attributed to a group of processes associated with the transfer of electrons between a molecule and substrate [40]. As stated, plasmons contribute strongly to electromagnetic enhancement when LSPR bands are in the visible spectral region, allowing for strong light absorption. As a result, a strong electromagnetic enhancement is observed from noble metals since their LSPR bands are in the visible spectra region. However, semiconductors usually have the LSPR peak of their VB centered in the UV region and the LSPR peak of the conduction band at the near-infrared spectra region. Hence, plasmons of semiconductors rarely contribute to electromagnetic enhancement [11]. When plasmonic substrates like Au are coated onto the ZnO substrate, the dual effects of electromagnetic enhancement from the plasmonic material and chemical enhancement resulting from charge transfer at the metal-semiconductor interface are harnessed [12–14]. To illustrate the charge transfer effect, the Fermi energy level of Au is given as −5.1 eV [23,28], while the lowest unoccupied molecular orbital (LUMO) of CV is −4.1 eV [30,41]. The energy difference between this Fermi energy level of Au and the conduction band (CB) of CV is less than the energy provided by the 785 nm laser. Thus, electrons can be transferred directly from the Fermi energy level of Au to the LUMO of CV. Also, ZnO has a CB at −4.3 eV [23], which can serve as a bridge for electron transfer between Au and CV. This is because the CB is between the Fermi energy level of Au and the LUMO of CV. Thus, electrons are transferred from the Fermi energy level of Au to the CB of ZnO and then to the LUMO of CV.

Additionally, the combined configuration allows a large surface area for plasmonic metals to be attached. This increases the possibility of forming hotspots and allows more molecules or target analytes to be attached to the substrate surface. To summarize, the enhanced Raman signal of the CV molecule observed with Au-ZnO substrates can be attributed to the following: (i) the electromagnetic effect provided by Au nanoparticles, (ii) the transfer of electrons between the Fermi energy level of Au, CB of ZnO, and LUMO of CV, and (iii) the large surface area provided by ZnO that allowed more hotspots to be formed and more CV to be attached. Earlier research on hybrid substrates emphasizes their advantages, which include better sensitivity, enhanced optical performance, higher stability, and signal reproducibility compared to plasmonic materials [11,35,42–44].

### 3.3. Exosome Isolation and Characterization

**Figure 6.**
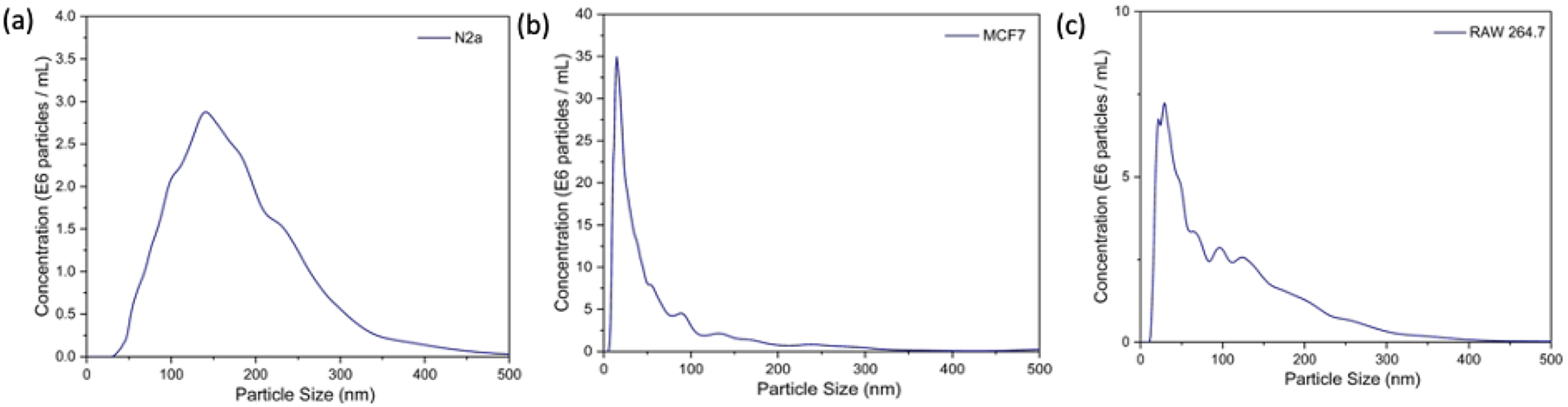
NTA results show the mean hydrodynamic diameter of exosomes isolated from (**a**) N2a cells, (**b**) MCF-7 cells, (**c**) RAW264.7 cells.

Exosomes were extracted from the conditioned media of MCF-7, N2a, and RAW 264.7 cells using a commercial precipitation kit (Total Exosome Isolation Reagent, Invitrogen, Waltham, MA, USA). As shown in Figure 6, the NTA results show the mean hydrodynamic diameter of exosomes isolated from N2a, MCF-7, and RAW264.7 as 181 nm, 88 nm, and 110 nm, respectively. The exosome concentrations in the N2a cell line, MCF-7 cells, and RAW264 were measured to be 4.71 × 10^8^ particles/mL, 13.36 × 10^8^ particles/mL, and 6.52 × 10^8^ particles/mL, respectively.

### 3.4. Exosome Detection

**Figure 7.**
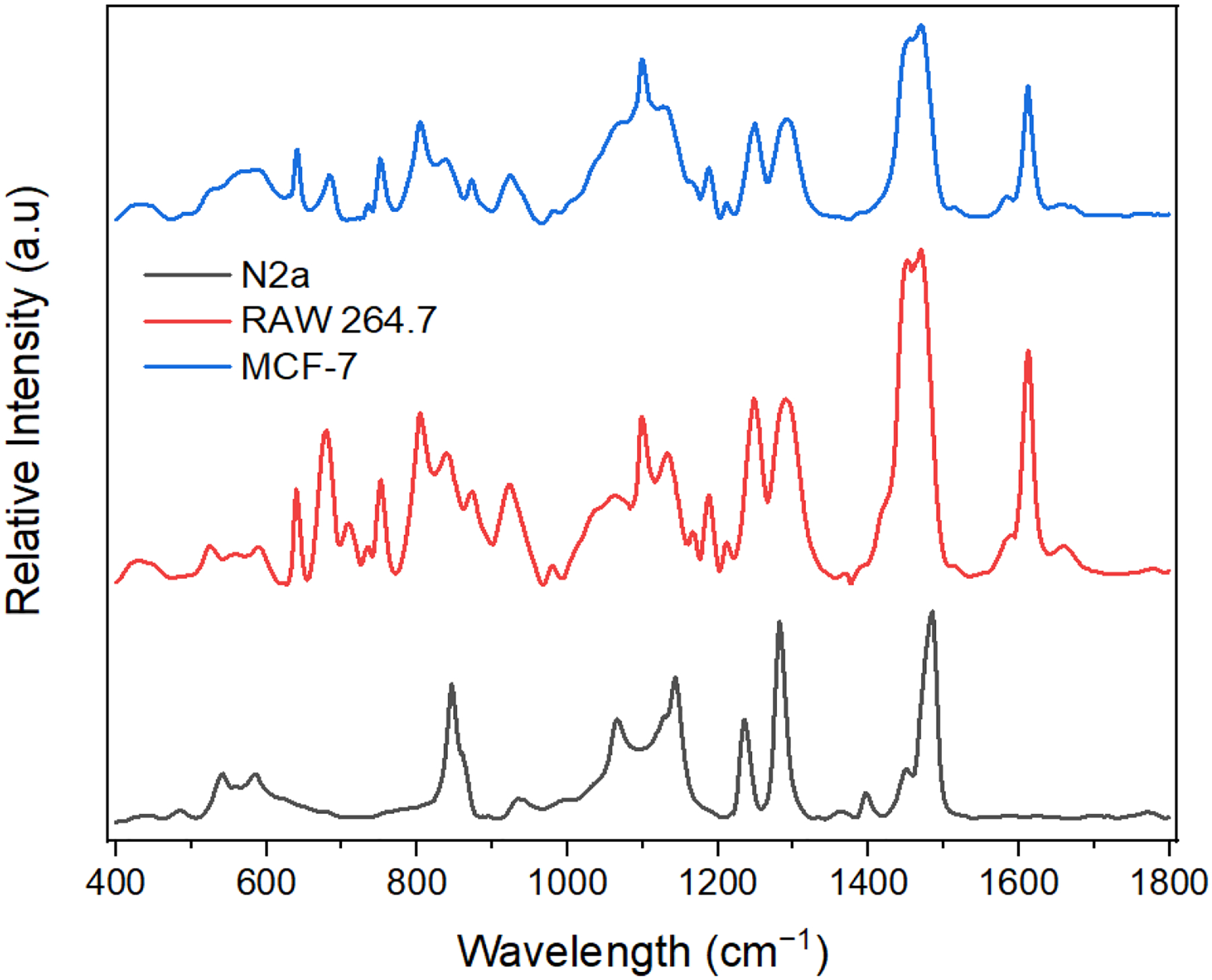
SERS spectra of different exosomes on the optimal substrate (ZnO-Au2).

The SERS technique reveals the vibrational modes of molecules with peaks corresponding to specific vibrational modes of molecules present in the sample. It can therefore indicate the biomolecules that are present in these exosomes. However, several factors, notably the sample preparation technique, the excitation wavelength, and the instrument used, might influence the SERS peaks. To test the biosensing capabilities of the optimal hybrid substrate, we chose three different exosomes derived from neuroblastoma cells (N2a), macrophages (RAW 264.7), and breast cancer cells (MCF-7). Through this system, we expected to uncover molecular variability between different exosome types (see Figure 7). These results also showed good reproducibility between measurements (see Supporting Information, Figures S1–S3).

As shown in Table 1, various peaks were obtained for the exosomes and assigned to different biomolecules. The macromolecules indicated by the SERS peaks include nucleic acids, proteins, lipids, and phospholipids [17,45–49]. Results show some common SERS peaks since exosomes contain similar macromolecule profiles. For example, the SERS spectra from the three exosomes indicated a peak at 1452 cm^−1^, attributed to δ(CH_2_, CH_3_) deformations in lipids and proteins [17,45]. However, different exosomes have unique fingerprints with characteristic SERS peaks and different peak intensities, indicating that exosomes from different cells exhibit some unique SERS features. For instance, tyrosine present at 1612 cm^−1^ in both MCF-7 and RAW 264.7 exosomes was absent in N2a, while the stretching mode ν(C_α_–N, C_α_–C, C–N) of protein’s backbone present in N2a exosomes at 1142 cm^−1^ was found in MCF-7 and RAW exosomes at 1133 cm^−1^ and 1130 cm^−1^, respectively. Furthermore, nucleic acid (purine A, G ring) was detected at 1470 cm^−1^ in MCF-7 and RAW 264.7 exosomes and was observed at 1484 cm^−1^ in N2a exosomes. In addition, the nucleic acids (pyrimidine and imidazole rings A/G stacking) present at 1397 cm^−1^ in N2a exosomes had a low SERS intensity at 1370 cm^−1^ and 1392 cm^−1^ in RAW 264.7 and MCF-7, respectively. Regarding lipids, the bending vibration δ(CH2) in the acyl chain was found at 1290 cm^−1^ and 1291 cm^−1^ in RAW 264.7 and MCF-7 exosomes, respectively, but at 1281 cm^−1^ in N2a exosomes. In summary, while the SERS spectra of exosomes from N2a, RAW 264.7, and MCF-7 cells exhibit some similarities, the differences may reflect variations in the biomolecular composition, biological function, and metabolic pathways, which suggests potential applications in exosome detection.

## 4. Conclusions

This study shows the potential of SERS in differential biological samples (exosomes) using a hybrid semiconductor substrate. Pure ZnO nanoparticles were synthesized and coated with different concentrations of HAuCl_4_ (0.01, 0.05, and 0.1 M) using a wet chemistry method. TEM and XPS analyses confirmed the successful synthesis of ZnO with Au. In the UV-Vis spectrum of Au-coated ZnO, a reduction in the peak of ZnO nanoparticles and an extra peak corresponding to Au were observed. The maximum SERS enhancement was obtained from ZnO coated with 0.05 M HAuCl_4_. An enhancement factor of 2.9 × 10^4^ was calculated for this substrate with an LOD of 10^−8^ of CV. Subsequently, exosomes from neuroblastoma cells (N2a), macrophages (RAW 264.7), and breast cancer cells (MCF-7) were selected to demonstrate molecular variability between different cell types. This work is expected to serve as a platform for SERS-based diagnostic applications for exosome detection and other 3D biological systems.

## Supplementary Material

Supplementary Materials

## Figures and Tables

**Table 1.** SERS peaks observed from exosome populations isolated from three cell types and corresponding assignments [17,45–4,9].

Neuroblastoma Cells (N2a)			Macrophages (RAW 264.7)			Breast Cancer Cells (MCF-7)		
Peak (cm^−1^)	Biomolecule	Assignment	Peak (cm^−1^)	Biomolecule	Assignment	Peak (cm^−1^)	Biomolecule	Assignment
1484	Nucleic acid	Purine A, G ring	1658	Lipid	ν(C = C) in acyl chain	1653	Lipid	ν(C = C) in acyl chain
1452	Protein	Backbone δ(CH2,CH3)	1612	Protein	Tyrosine	1612	Protein	Tyrosine
	Lipid	δ(CH2, CH3) in acyl chain						
1397	Nucleic acid	Pyrimidine and imidazole rings A/G stacking	1470	Nucleic acid	Purine A, G ring	1470	Nucleic acid	Purine A, G ring
1362	Protein	Tryptophan	1452	Protein	Backbone δ(CH2, CH3)	1452	Proteins	Backbone δ(CH2, CH3)
				Lipid	δ(CH2, CH3) in acyl chain		Lipid	δ(CH2, CH3) in acyl chain
1281	Lipid	δ(CH2) in acyl chain	1370	Nucleic acid	Pyrimidine and imidazole rings A/G stacking	1392	Nucleic acid	Pyrimidine and imidazole rings A/G stacking
1232	Protein	Amide III: ν(C–N) + δ(NH)	1290	Lipid	δ(CH2) in acyl chain	1291	Lipid	δ(CH2) in acyl chain
1142	Protein	Backbone ν(C_*α*_–N, C_*α*_–C, C–N)	1247	Protein	AmideIII: ν(C–N) + δ(NH)	1249	Protein	AmideIII: ν(C–N) + δ(NH)
	Lipid	ν(C–C) in acyl chain						
1065	Lipid	ν(C–C)	1211	Protein	Phenylalanine, Tyrosine	1210	Protein	Phenylalanine, Tyrosine
932	Protein	*α*-Helix backbone ν(C–C_*α*_–N)	1187	Protein	Tyrosine	1187	Protein	Tyrosine
860	Phospholipid	ν(O–C–C–N^+^), ν(C_4_–N^+^)	1130	Protein	Backbone ν(C_*α*_–N, C_*α*_–C, C–N)	1133	Protein	Backbone ν(C_*α*_–N, C_*α*_–C, C–N)
				Lipid	ν(C–C) in acyl chain		Lipid	ν(C–C) in acyl chain
846	Protein	Tyrosine (Y6)	1098	Nucleic acid	Phosphodioxy ν_s_(PO_2_^–^)	1098	Nucleic acid	Phosphodioxy ν_s_(PO_2_^–^)
			1063	Lipid	ν(C–C)	1072	Lipid	ν(C–C)
			922	Protein	*α*-Helix	923	Protein	*α*-Helix backbone ν(C–C_*α*_–N)
					backbone ν(C–C_*α*_–N)			
			873	Phospholipid	ν(O–C–C–N+), ν(C4–N+)	872	Phospholipid	ν(O–C–C–N+), ν(C4–N+)
			838	Protein	Tyrosine (Y6)	836	Protein	Tyrosine (Y6)
			804	Nucleic acid	Phosphodiester ν_s_(O–P–O)	804	Nucleic acid	Phosphodiester ν_s_(O–P–O)
			751		ν (pyrrole breathing)	751		ν(pyrrole breathing)
			707	Lipids	Cholesterol	734	Nucleic acid	Adenine
			679	Nucleic acid	Guanine	683	Nucleic acid	Guanine

ν = Stretching mode, δ = deformation mode.

## Data Availability

Data are available within this article.
