# Supplementary material for: Au-Coated ZnO Surface-Enhanced Raman Scattering (SERS) Substrates: Synthesis, Characterization, and Applications in Exosome Detection
Source: Chemosensors (Basel). Author manuscript; Available in PMC 2024 Oct 4. (PMC11450680; doi:10.3390/chemosensors11110554)
Supplement: Supplementary Materials [file NIHMS1977421-supplement-Supplementary_Materials.pdf]

<u>**Supporting Information**</u>

Au-coated ZnO Surface-Enhanced Raman Scattering (SERS) Substrates: Synthesis, Characterization, and Applications in Exosome Detection

Samuel Adesoye [1], Saqer Al Abdullah [1], Anjali Kumari [1], Gayani Pathiraja [2], Kyle Nowlin [2] and Kristen Dellinger [1,*]

[1] Department of Nanoengineering, Joint School of Nanoscience and Nanoengineering, North Carolina A&T State University, 2907 E Gate City Blvd, Greensboro, NC 27401, USA; soadesoye@aggies.ncat.edu (S.A.); smalabdullah@aggies.ncat.edu (S.A.A.); akumari@aggies.ncat.edu (A.K.)

[2] Department of Nanoscience, Joint School of Nanoscience and Nanoengineering, University of North Carolina at Greensboro, 2907 E Gate City Blvd, Greensboro, NC 27401, USA; gcpathir@uncg.edu (G.P.); ksnowlin@uncg.edu (K.N.)

* Correspondence: kdellinger@ncat.edu

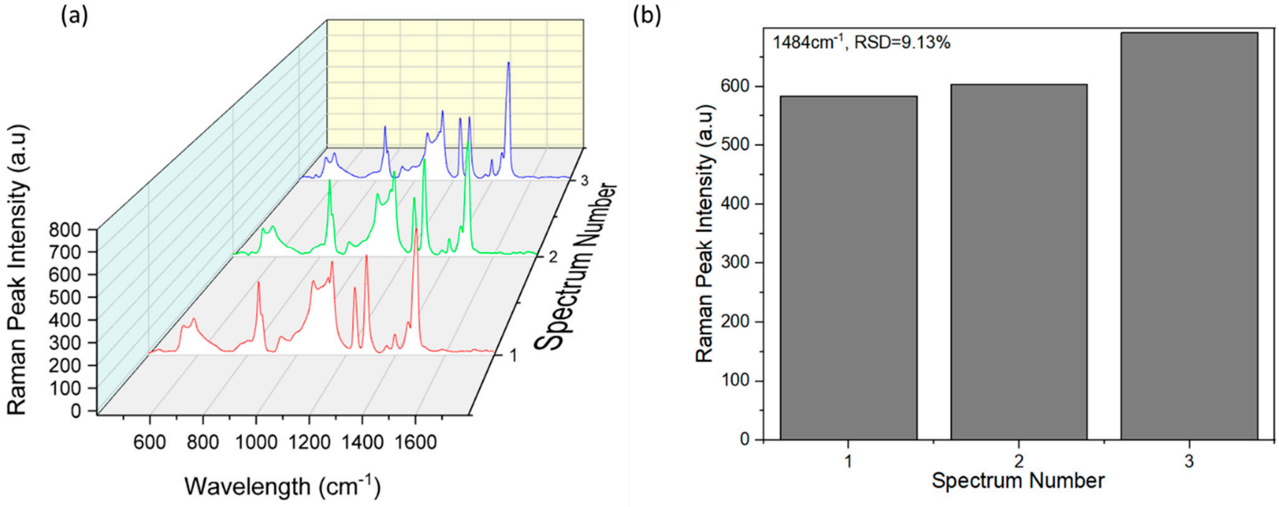

Figure S1. (a) SERS spectra of three random measurements of N2a on the optimal substrate (b) SERS peak intensity at wavelength 1484cm^{-1} of the three random measurements of N2a on the optimal substrate.

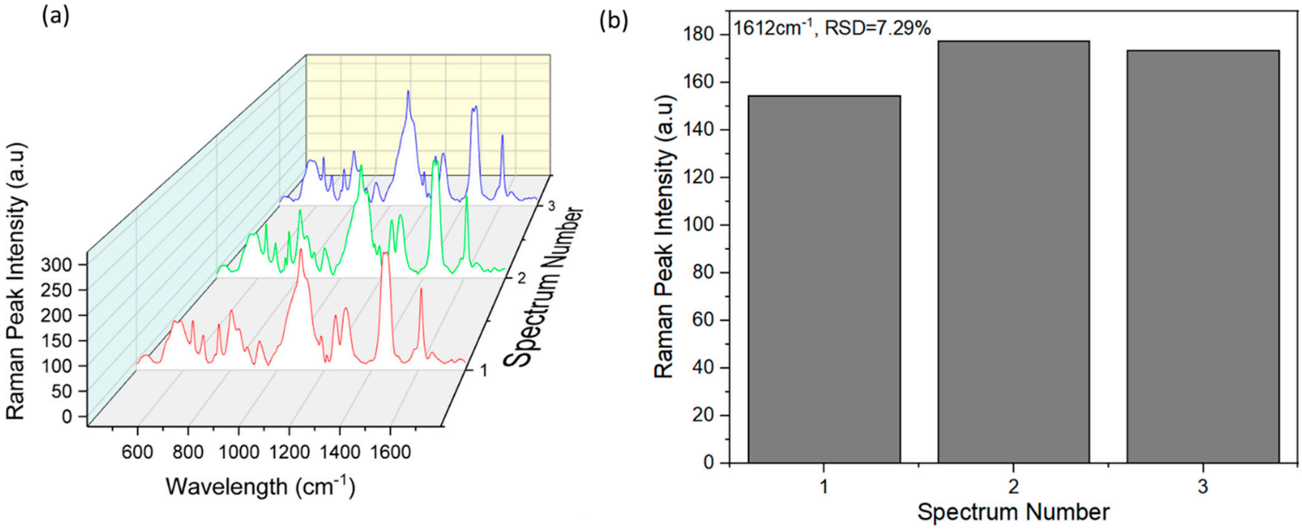

Figure S2. (a) SERS spectra of three random measurements of RAW 264.7 on the optimal substrate (b) SERS peak intensity at wavelength 1612cm^{-1} of the three random measurements of RAW 264.7 on the optimal substrate.

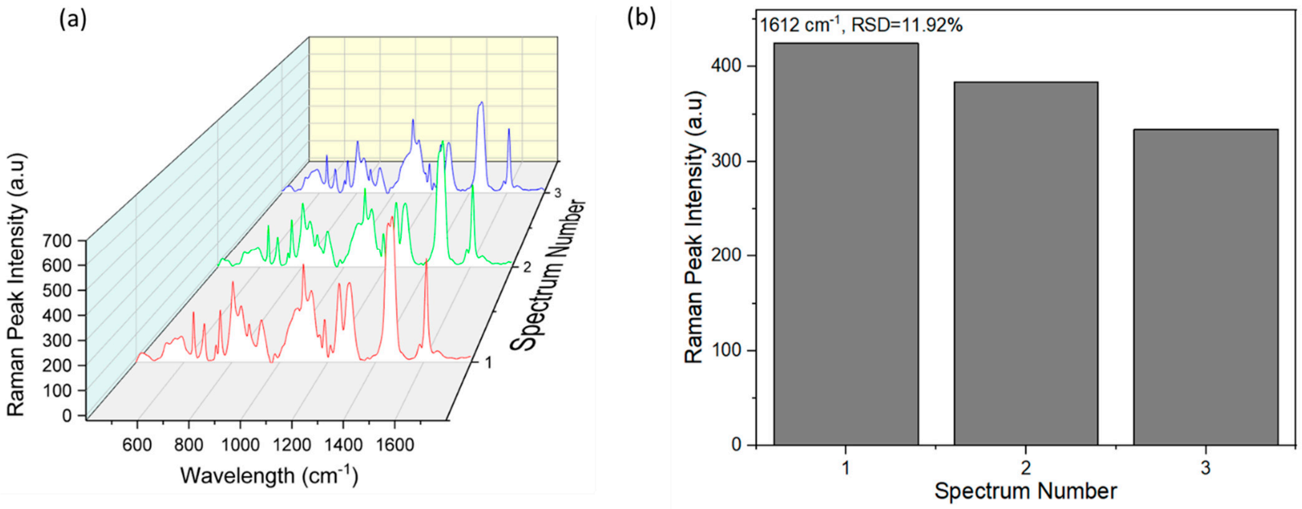

Figure S3. (a) SERS spectra of three random measurements of MCF-7 on the optimal substrate (b) SERS peak intensity at wavelength 1612 cm^{-1} of the three random measurements of MCF-7 on the optimal substrate.
